# Supplementary material for: Explaining detection heterogeneity with finite mixture and non-Euclidean movement in spatially explicit capture-recapture models
Source: PeerJ. 2022 Jun 7;10:e13490. doi: 10.7717/peerj.13490 (PMC9186326; doi:10.7717/peerj.13490)
Supplement: Supplemental Information 1 — The area was the region of the convex hull that surrounded the bear hair corrals of each study area. The mask area was the region of integration of the spatially explicit capture-recapture model. Linear feature density is the number of 10-meter pixels within a 30-meter pixel that contained a road (At most 9 pixels). Water body density is the number of 15-meter pixels within a 30-meter pixel that contained a waterbody (At most 4 pixels). μ is the mean, R is the range, and σ is the standard deviation. GLSL is the Great Lakes-St Lawrence Forest region. [file peerj-10-13490-s001.docx]

| **Trapline** | **Year** | **Forest Region** | **Areas (km^2^)** | | **Elevation (meters)** | | | | **Vector Ruggedness Measure** | | | | **Linear feature density** | | | | **Waterbody density** | | |
| --- | --- | --- | --- | --- | --- | --- | --- | --- | --- | --- | --- | --- | --- | --- | --- | --- | --- | --- | --- |
|  |  |  | **Trapline** | **Mask** | **µ** | ***R*** | **σ** | **µ** | | ***R*** | **σ** | **µ** | | ***R*** | **σ** | **µ** | | ***R*** | **σ** |
| 325Road | 2017 | Boreal | 341.4 | 2391.9 | 420.8 | 155.6 | 21.2 | 0.0002 | | 0.0319 | 0.0004 | 0.076 | | 9.000 | 0.525 | 0.894 | | 4.000 | 1.629 |
| 700Rd | 2017 | GLSL | 338.6 | 2276.5 | 424.4 | 224.0 | 24.5 | 0.0008 | | 0.0431 | 0.0013 | 0.076 | | 9.000 | 0.525 | 0.468 | | 4.000 | 1.248 |
| 81Rd | 2017 | Boreal | 178.9 | 2680.3 | 400.9 | 403.7 | 92.1 | 0.0005 | | 0.0610 | 0.0011 | 0.118 | | 8.000 | 0.643 | 0.679 | | 4.000 | 1.447 |
| BeautyLakeRoad | 2018 | Boreal | 333.4 | 2372.7 | 400.4 | 390.5 | 50.2 | 0.0004 | | 0.0502 | 0.0009 | 0.111 | | 9.000 | 0.622 | 0.329 | | 4.000 | 1.075 |
| Borland | 2017 | Boreal | 329.1 | 2578.8 | 402.1 | 266.8 | 36.8 | 0.0002 | | 0.0239 | 0.0004 | 0.169 | | 9.000 | 0.779 | 0.353 | | 4.000 | 1.106 |
| Caithness | 2018 | Boreal | 312.4 | 2387.4 | 319.9 | 201.4 | 27.8 | 0.0001 | | 0.0097 | 0.0001 | 0.165 | | 9.000 | 0.759 | 0.175 | | 4.000 | 0.742 |
| Camp1Road | 2018 | Boreal | 298.9 | 2485.9 | 389.5 | 122.9 | 26.5 | 0.0004 | | 0.0305 | 0.0007 | 0.087 | | 9.000 | 0.583 | 1.105 | | 4.000 | 1.762 |
| Cargill | 2018 | Boreal | 314.3 | 2617.8 | 256.9 | 104.1 | 17.8 | 0.0001 | | 0.0051 | 0.0001 | 0.244 | | 9.000 | 0.906 | 0.198 | | 4.000 | 0.831 |
| CCGP | 2017 | Boreal | 337.9 | 2570.3 | 406.7 | 314.6 | 42.5 | 0.0002 | | 0.0151 | 0.0004 | 0.136 | | 9.000 | 0.697 | 0.387 | | 4.000 | 1.154 |
| CedarNarrows | 2018 | GLSL | 507.3 | 2677.4 | 391.9 | 151.7 | 23.9 | 0.0007 | | 0.0547 | 0.0011 | 0.049 | | 9.000 | 0.439 | 0.976 | | 4.000 | 1.680 |
| CenturyRoad | 2018 | Boreal | 231.9 | 2436.7 | 385.8 | 138.2 | 21.6 | 0.0005 | | 0.0547 | 0.0008 | 0.066 | | 9.000 | 0.504 | 0.970 | | 4.000 | 1.683 |
| CribRoad | 2017 | Boreal | 265.8 | 2471.0 | 337.9 | 128.1 | 14.4 | 0.0001 | | 0.0111 | 0.0002 | 0.116 | | 8.000 | 0.638 | 0.440 | | 4.000 | 1.201 |
| DeerLakeRoad | 2018 | Boreal | 253.6 | 2293.9 | 370.0 | 166.9 | 29.7 | 0.0007 | | 0.0288 | 0.0011 | 0.079 | | 9.000 | 0.555 | 0.792 | | 4.000 | 1.567 |
| Detour | 2018 | Boreal | 141.8 | 2522.1 | 297.5 | 157.7 | 14.8 | 0.0000 | | 0.0366 | 0.0001 | 0.067 | | 8.000 | 0.486 | 0.422 | | 4.000 | 1.175 |
| FredFlat | 2018 | Boreal | 411.5 | 2573.2 | 218.8 | 139.8 | 13.4 | 0.0000 | | 0.0123 | 0.0001 | 0.181 | | 9.000 | 0.795 | 0.277 | | 4.000 | 0.972 |
| Fushimi | 2018 | Boreal | 281.0 | 2350.3 | 208.3 | 200.3 | 41.2 | 0.0001 | | 0.0143 | 0.0002 | 0.143 | | 8.000 | 0.707 | 0.264 | | 4.000 | 0.924 |
| GardenLakeRoad | 2017 | Boreal | 281.0 | 2459.6 | 455.5 | 218.7 | 27.2 | 0.0002 | | 0.0202 | 0.0004 | 0.111 | | 9.000 | 0.642 | 0.458 | | 4.000 | 1.213 |
| GargMijnSand | 2018 | GLSL | 261.2 | 2431.5 | 344.4 | 444.2 | 97.7 | 0.0009 | | 0.1154 | 0.0016 | 0.017 | | 8.000 | 0.248 | 1.008 | | 4.000 | 1.720 |
| GibsonLakeRd | 2017 | Boreal | 265.5 | 2172.4 | 306.3 | 214.6 | 30.2 | 0.0001 | | 0.0181 | 0.0003 | 0.104 | | 8.000 | 0.603 | 0.408 | | 4.000 | 1.194 |
| GoldfieldRoad | 2017 | Boreal | 288.4 | 2650.2 | 390.2 | 220.7 | 37.8 | 0.0003 | | 0.0331 | 0.0007 | 0.135 | | 9.000 | 0.685 | 0.517 | | 4.000 | 1.287 |
| Grassy | 2017 | Boreal | 194.5 | 2652.4 | 363.1 | 199.9 | 17.6 | 0.0003 | | 0.0405 | 0.0005 | 0.104 | | 8.000 | 0.603 | 0.426 | | 4.000 | 1.201 |
| Hwy651 | 2017 | Boreal | 300.4 | 2331.5 | 404.2 | 285.7 | 46.8 | 0.0005 | | 0.0386 | 0.0009 | 0.079 | | 9.000 | 0.536 | 0.540 | | 4.000 | 1.332 |
| InglisLakeRoad | 2018 | Boreal | 276.5 | 2483.5 | 264.9 | 169.4 | 13.6 | 0.0001 | | 0.1441 | 0.0002 | 0.072 | | 9.000 | 0.500 | 0.340 | | 4.000 | 1.052 |
| LarderRaven | 2017 | Boreal | 305.0 | 1837.9 | 315.4 | 291.9 | 28.8 | 0.0006 | | 0.0711 | 0.0011 | 0.096 | | 9.000 | 0.582 | 0.338 | | 4.000 | 1.092 |
| Longlegged | 2018 | Boreal | 424.2 | 2451.6 | 395.1 | 113.0 | 21.8 | 0.0006 | | 0.0360 | 0.0009 | 0.042 | | 9.000 | 0.395 | 0.919 | | 4.000 | 1.650 |
| MasseyTote | 2018 | GLSL | 312.9 | 2423.1 | 400.2 | 327.5 | 57.8 | 0.0011 | | 0.1278 | 0.0015 | 0.074 | | 9.000 | 0.518 | 0.336 | | 4.000 | 1.077 |
| McConnell | 2017 | GLSL | 361.8 | 2150.8 | 326.0 | 305.6 | 39.4 | 0.0003 | | 0.0378 | 0.0007 | 0.143 | | 9.000 | 0.734 | 0.315 | | 4.000 | 1.054 |
| MenetBrent | 2018 | GLSL | 219.2 | 1859.0 | 298.1 | 393.9 | 77.0 | 0.0006 | | 0.0648 | 0.0009 | 0.130 | | 9.000 | 0.682 | 0.344 | | 4.000 | 1.098 |
| MunroTower | 2017 | Boreal | 295.6 | 2739.8 | 300.5 | 232.2 | 26.3 | 0.0002 | | 0.0711 | 0.0004 | 0.123 | | 9.000 | 0.652 | 0.404 | | 4.000 | 1.186 |
| Opeepeesway | 2017 | Boreal | 238.8 | 2050.2 | 412.0 | 186.1 | 21.9 | 0.0002 | | 0.0197 | 0.0004 | 0.142 | | 9.000 | 0.742 | 0.373 | | 4.000 | 1.139 |
| OpeongoLine | 2018 | GLSL | 534.6 | 2736.2 | 412.3 | 402.3 | 64.0 | 0.0009 | | 0.0497 | 0.0012 | 0.112 | | 9.000 | 0.631 | 0.414 | | 4.000 | 1.191 |
| Pardo | 2017 | GLSL | 373.6 | 2528.9 | 297.1 | 223.6 | 34.8 | 0.0006 | | 0.0855 | 0.0010 | 0.121 | | 9.000 | 0.673 | 0.471 | | 4.000 | 1.258 |
| Pineridge | 2018 | Boreal | 295.3 | 2442.9 | 400.0 | 136.8 | 22.9 | 0.0003 | | 0.0146 | 0.0005 | 0.079 | | 8.000 | 0.532 | 0.982 | | 4.000 | 1.686 |
| PortelanceRd | 2017 | GLSL | 238.6 | 2330.4 | 372.7 | 426.8 | 53.9 | 0.0008 | | 0.0773 | 0.0013 | 0.070 | | 8.000 | 0.502 | 0.566 | | 4.000 | 1.362 |
| RedSquirrel | 2018 | GLSL | 380.6 | 2386.4 | 324.7 | 341.4 | 41.4 | 0.0006 | | 0.0294 | 0.0009 | 0.100 | | 9.000 | 0.618 | 0.720 | | 4.000 | 1.506 |
| RobinsonLake | 2018 | Boreal | 321.5 | 2323.1 | 461.8 | 200.3 | 26.4 | 0.0003 | | 0.0248 | 0.0006 | 0.151 | | 9.000 | 0.753 | 0.346 | | 4.000 | 1.100 |
| RoundLake | 2018 | GLSL | 352.1 | 2416.4 | 262.8 | 399.1 | 83.3 | 0.0007 | | 0.0549 | 0.0011 | 0.152 | | 8.000 | 0.730 | 0.374 | | 4.000 | 1.143 |
| ShirleyLakeRd | 2018 | GLSL | 360.6 | 2462.2 | 400.0 | 365.3 | 52.4 | 0.0007 | | 0.0426 | 0.0011 | 0.025 | | 9.000 | 0.302 | 0.532 | | 4.000 | 1.335 |
| SouthEMURd | 2017 | Boreal | 189.1 | 1959.1 | 310.7 | 228.8 | 24.4 | 0.0003 | | 0.0266 | 0.0006 | 0.166 | | 9.000 | 0.752 | 0.161 | | 4.000 | 0.765 |
| SowdenMckenzie | 2017 | Boreal | 194.2 | 2091.1 | 451.4 | 129.8 | 20.1 | 0.0001 | | 0.0187 | 0.0003 | 0.095 | | 9.000 | 0.579 | 0.493 | | 4.000 | 1.274 |
| SowdenLine | 2018 | Boreal | 181.0 | 1980.6 | 453.3 | 129.8 | 19.8 | 0.0001 | | 0.0187 | 0.0003 | 0.096 | | 9.000 | 0.583 | 0.491 | | 4.000 | 1.272 |
| Translimit | 2018 | Boreal | 263.9 | 2456.6 | 305.4 | 176.8 | 21.1 | 0.0001 | | 0.0078 | 0.0002 | 0.049 | | 8.000 | 0.414 | 0.256 | | 4.000 | 0.926 |
| TurtleRiverRoad | 2018 | GLSL | 210.0 | 2320.4 | 381.2 | 149.1 | 24.6 | 0.0004 | | 0.0226 | 0.0007 | 0.051 | | 9.000 | 0.441 | 0.719 | | 4.000 | 1.511 |
| VermilionRiverRoad | 2018 | Boreal | 257.0 | 2231.4 | 391.0 | 123.7 | 21.4 | 0.0005 | | 0.0245 | 0.0008 | 0.101 | | 9.000 | 0.619 | 0.718 | | 4.000 | 1.465 |
| WatabeagRd | 2017 | Boreal | 411.6 | 2683.6 | 320.2 | 224.7 | 32.3 | 0.0002 | | 0.0190 | 0.0005 | 0.146 | | 9.000 | 0.709 | 0.231 | | 4.000 | 0.911 |
| Wenasaga | 2018 | Boreal | 204.6 | 2597.9 | 395.4 | 136.3 | 23.4 | 0.0003 | | 0.0240 | 0.0005 | 0.084 | | 8.000 | 0.543 | 0.585 | | 4.000 | 1.384 |
| Wenebegon | 2018 | Boreal | 328.6 | 2557.6 | 464.2 | 280.1 | 32.9 | 0.0005 | | 0.0432 | 0.0010 | 0.159 | | 9.000 | 0.768 | 0.282 | | 4.000 | 0.996 |
| WhitmanDam | 2018 | GLSL | 239.3 | 2275.6 | 444.0 | 427.9 | 63.5 | 0.0012 | | 0.0799 | 0.0018 | 0.077 | | 9.000 | 0.529 | 0.296 | | 4.000 | 1.013 |
| WinterLake | 2018 | Boreal | 427.7 | 2669.9 | 302.1 | 165.1 | 20.1 | 0.0001 | | 0.0181 | 0.0002 | 0.086 | | 8.000 | 0.547 | 0.127 | | 4.000 | 0.684 |
| WMU54Line | 2018 | GLSL | 358.8 | 2359.7 | 413.0 | 254.8 | 56.3 | 0.0010 | | 0.0360 | 0.0014 | 0.092 | | 9.000 | 0.576 | 0.693 | | 4.000 | 1.482 |
| WMU50ParrySound | 2018 | GLSL | 319.0 | 2508.0 | 416.9 | 310.3 | 51.4 | 0.0010 | | 0.0360 | 0.0013 | 0.081 | | 9.000 | 0.542 | 0.352 | | 4.000 | 1.107 |
| **Average** | | | **300.9** | **2405.8** | **350.8** | **633.2** | **86.5** | **0.0004** | | **0.1500** | **0.0009** | **0.100** | | **9.000** | **0.601** | **0.518** | | **4.000** | **1.309** |
