# Supplementary material for: Explaining detection heterogeneity with finite mixture and non-Euclidean movement in spatially explicit capture-recapture models
Source: PeerJ. 2022 Jun 7;10:e13490. doi: 10.7717/peerj.13490 (PMC9186326; doi:10.7717/peerj.13490)
Supplement: Supplemental Information 2 — MMDM refers to Mean Maximum Distance Moved and is the average maximum distance between detections of each black bear. d is the mean distance between consecutive capture locations pooled over individuals. [file peerj-10-13490-s002.docx]

| **Study area** | **Year** | **Samples** | **Genotypes** | **Captured** | **Detections** | **Mean DPA** | **Movements** | **MMDM** | $\bar{\boldsymbol{d}}$ |
| --- | --- | --- | --- | --- | --- | --- | --- | --- | --- |
| 325Road | 2017 | 576 | 448 | 48 | 131 | 2.73 | 69 | 3989.72 | 2490.19 |
| 700Rd | 2017 | 399 | 311 | 34 | 95 | 2.79 | 46 | 2467.82 | 1538.54 |
| 81Rd | 2017 | 1522 | 1156 | 67 | 350 | 5.22 | 254 | 5989.83 | 2712.77 |
| BeautyLakeRoad | 2018 | 658 | 529 | 44 | 143 | 3.25 | 83 | 3550.28 | 2720.70 |
| Borland | 2017 | 507 | 363 | 32 | 145 | 4.53 | 106 | 6096.17 | 2800.34 |
| Caithness | 2018 | 530 | 373 | 38 | 145 | 3.82 | 103 | 5371.30 | 3416.85 |
| Camp1Road | 2018 | 921 | 679 | 64 | 217 | 3.39 | 138 | 6432.79 | 3962.43 |
| Cargill | 2018 | 422 | 352 | 61 | 128 | 2.10 | 41 | 3961.82 | 2764.07 |
| CCGP | 2017 | 1152 | 908 | 61 | 319 | 5.23 | 241 | 5592.71 | 2586.66 |
| CedarNarrows | 2018 | 1181 | 920 | 75 | 253 | 3.37 | 138 | 2972.10 | 2180.09 |
| CenturyRoad | 2018 | 791 | 570 | 67 | 176 | 2.63 | 81 | 2414.87 | 2092.66 |
| CribRoad | 2017 | 1566 | 1035 | 69 | 315 | 4.57 | 227 | 6091.32 | 3196.10 |
| DeerLakeRoad | 2018 | 683 | 501 | 51 | 155 | 3.04 | 80 | 3519.95 | 2065.54 |
| Detour | 2018 | 740 | 549 | 37 | 193 | 5.22 | 147 | 5458.36 | 2626.87 |
| FredFlat | 2018 | 328 | 285 | 33 | 96 | 2.91 | 47 | 3943.24 | 2603.71 |
| Fushimi | 2018 | 682 | 529 | 51 | 173 | 3.39 | 114 | 4349.42 | 3056.98 |
| GardenLakeRoad | 2017 | 1199 | 983 | 71 | 293 | 4.13 | 201 | 4092.02 | 2375.15 |
| GargMijnSand | 2018 | 741 | 591 | 35 | 170 | 4.86 | 121 | 7523.03 | 3666.76 |
| GibsonLakeRd | 2017 | 604 | 456 | 53 | 160 | 3.02 | 89 | 3201.70 | 2315.71 |
| GoldfieldRoad | 2017 | 773 | 600 | 53 | 165 | 3.11 | 92 | 4111.16 | 2443.98 |
| Grassy | 2017 | 639 | 491 | 51 | 196 | 3.84 | 122 | 4755.00 | 2847.14 |
| Hwy651 | 2017 | 643 | 421 | 39 | 136 | 3.49 | 84 | 4314.34 | 2503.45 |
| InglisLakeRoad | 2018 | 847 | 629 | 52 | 194 | 3.73 | 131 | 8716.43 | 3900.45 |
| LarderRaven | 2017 | 128 | 93 | 23 | 38 | 1.65 | 10 | 1281.74 | 1500.85 |
| Longlegged | 2018 | 1023 | 703 | 82 | 246 | 3.00 | 144 | 4569.98 | 2812.69 |
| MasseyTote | 2018 | 270 | 210 | 24 | 57 | 2.38 | 28 | 4912.87 | 3023.50 |
| McConnell | 2017 | 480 | 332 | 53 | 118 | 2.23 | 51 | 3301.46 | 2485.65 |
| MenetBrent | 2018 | 807 | 571 | 81 | 190 | 2.35 | 85 | 3020.71 | 2075.62 |
| MunroTower | 2017 | 432 | 312 | 45 | 125 | 2.78 | 61 | 3104.38 | 1975.06 |
| Opeepeesway | 2017 | 592 | 438 | 42 | 142 | 3.38 | 90 | 4832.07 | 3066.41 |
| OpeongoLine | 2018 | 143 | 110 | 25 | 44 | 1.76 | 10 | 2308.49 | 1661.78 |
| Pardo | 2017 | 562 | 401 | 61 | 146 | 2.39 | 60 | 2374.59 | 1860.87 |
| Pineridge | 2018 | 604 | 409 | 52 | 132 | 2.54 | 66 | 4372.86 | 2858.34 |
| PortelanceRd | 2017 | 531 | 354 | 57 | 144 | 2.53 | 74 | 4704.41 | 2990.32 |
| RedSquirrel | 2018 | 449 | 330 | 42 | 94 | 2.24 | 44 | 3626.51 | 2693.16 |
| RobinsonLake | 2018 | 273 | 206 | 27 | 68 | 2.52 | 29 | 2940.87 | 2697.24 |
| RoundLake | 2018 | 317 | 219 | 44 | 72 | 1.64 | 16 | 1602.17 | 1259.86 |
| ShirleyLakeRd | 2018 | 579 | 465 | 59 | 141 | 2.39 | 65 | 2908.44 | 2158.76 |
| SouthEMURd | 2017 | 590 | 465 | 49 | 137 | 2.80 | 65 | 2393.93 | 1841.77 |
| SowdenMckenzie | 2017 | 596 | 435 | 40 | 148 | 3.70 | 94 | 4181.71 | 2721.23 |
| SowdenLine | 2018 | 992 | 721 | 48 | 240 | 5.00 | 186 | 5722.68 | 2928.18 |
| Translimit | 2018 | 1038 | 751 | 58 | 251 | 4.33 | 178 | 6564.50 | 3246.83 |
| TurtleRiverRoad | 2018 | 817 | 545 | 62 | 139 | 2.24 | 53 | 2319.08 | 1950.15 |
| VermilionRiverRoad | 2018 | 1224 | 926 | 74 | 307 | 4.15 | 212 | 5491.20 | 2900.14 |
| WatabeagRd | 2017 | 458 | 366 | 38 | 108 | 2.84 | 55 | 3012.39 | 1962.82 |
| Wenasaga | 2018 | 934 | 697 | 49 | 251 | 5.12 | 189 | 6191.82 | 3155.89 |
| Wenebegon | 2018 | 533 | 427 | 46 | 133 | 2.89 | 70 | 4276.62 | 2797.96 |
| WhitmanDam | 2018 | 681 | 556 | 37 | 190 | 5.14 | 142 | 7656.99 | 3992.73 |
| WinterLake | 2018 | 363 | 272 | 51 | 101 | 1.98 | 44 | 3401.16 | 2449.91 |
| WMU54Line | 2018 | 367 | 258 | 40 | 77 | 1.93 | 20 | 3334.28 | 2367.91 |
| WMU50ParrySound | 2018 | 348 | 283 | 39 | 72 | 1.85 | 20 | 2078.25 | 2040.93 |
|  |  | **34235** | **25534** | **2534** | **8259** | **3.22** | **4916** | **4223.56** | **2594.97** |
