# Supplementary figures and images for: Explaining detection heterogeneity with finite mixture and non-Euclidean movement in spatially explicit capture-recapture models
Source: PeerJ. 2022 Jun 7;10:e13490. doi: 10.7717/peerj.13490 (PMC9186326; doi:10.7717/peerj.13490)

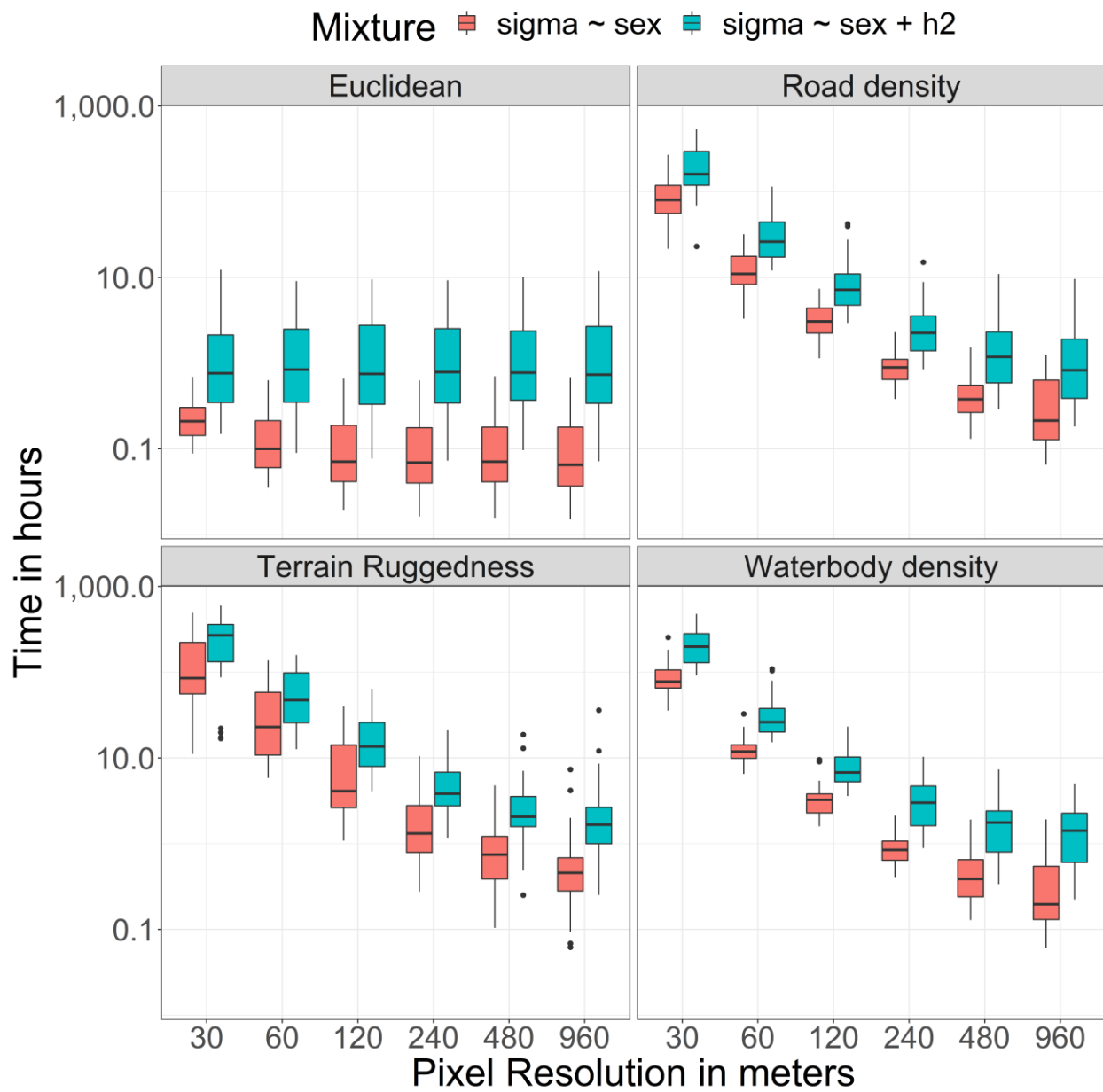

Supplement: Supplemental Information 3 [file peerj-10-13490-s003.pdf]
